# COVID-19 vaccination acceptance and trust among adults in Makkah, Saudi Arabia: a cross-sectional study

**DOI:** 10.1186/s42506-022-00116-2

**Published:** 2022-09-26

**Authors:** Mohamed O. Nour, Hatim A. Natto

**Affiliations:** 1 Department of Public Health and Community Medicine, Damietta Faculty of Medicine, Al-Azhar University, Damietta, Egypt; 2 Department of Health Education and Promotion, Faculty of Public Health & Health Informatics, Umm Al-Qura University, Makkah, Saudi Arabia; 3 Department of Epidemiology, Faculty of Public Health & Health Informatics, Umm Al-Qura University, Makkah, Saudi Arabia

**Keywords:** COVID-19, Vaccine, Knowledge, Acceptance, Trust

## Abstract

**Background:**

Public acceptance, trust, and actual uptake of COVID-19 vaccines are crucial to stem the pandemic. Although roll out of vaccines was high in KSA, the public response was not sufficiently studied. We aimed to investigate knowledge level, acceptance, and trust in COVID-19 vaccination and related predictors among adults in Makkah, KSA.

**Methods:**

A web-based cross-sectional survey using a snowballing sample was carried on 507 adult Saudi population living in Makkah city. The survey was developed based on literature search. In the logistic analysis, the dependent variables included acceptance rate and trust in effectiveness and safety of COVID-19 vaccines, while the independent variables (predictors) were sociodemographics and level of knowledge.

**Results:**

The survey included 507 participants, aged 18–78 years, 55.8% were females, and 36.7% had (or one of their family members) previously been exposed to COVID-19 infection. Their knowledge about COVID-19 vaccination was satisfactory (86.2%) with 71.2% intended to receive COVID-19 vaccination, and 56.4% was confident of the vaccine effectiveness. Vaccine efficacy, duration of protection, schedule of vaccination, and recommendation by authorities may favor their decision to accept or decline COVID-19 vaccines. Good knowledge about vaccines (*OR* = 2.07; *CI*: 1.24–3.48 for acceptance and *OR* = 2.67; *CI*: 1.58–4.51 for trust), higher educational level (*OR* = 1.80; *CI*: 1.07–3.40 for acceptance and *OR* = 3.59; *CI*: 2.08–6.21 for trust), previous seasonal flu vaccination (*OR* = 1.66; *CI*: 1.09–2.53 for acceptance and *OR* = 1.91; *CI*: 1.31–2.79 for trust), female sex (*OR* = 1.62; *CI*: 1.1–2.39 for acceptance and *OR* = 4.15; *CI*: 2.86–6.04 for trust), and history of COVID-19 infection (*OR* = 1.57; *CI*: 1.04–2.37 for acceptance and *OR* = 1.69; *CI*: 1.17–2.46 for trust) were among significant predictors for both vaccine acceptance and trust in vaccine effectiveness.

**Conclusions:**

Adult Saudi population in Makkah city showed satisfactory knowledge about COVID-19 vaccination with moderate rate of vaccine acceptance and a relatively low rate of confidence in vaccine effectiveness. Better understanding of public acceptance and trust in COVID-19 vaccines and addressing barriers to vaccination are recommended to improve vaccine coverage and to reinforce some communication characteristics of the current vaccination campaign.

## Introduction

The world is currently facing one of the biggest challenges, the coronavirus disease (COVID-19), which rapidly spread all over the world within a very short period to be declared by the World Health Organization (WHO) a worldwide pandemic. It led to economic loses, social ramifications, and a heavy load on healthcare sector [1]. The WHO situation report on April 1, 2022, confirmed over 486 million cases with about 6.14 million deaths globally. Cases officially confirmed in Saudi Arabia were 750,814 cases with 9045 deaths reported, and the number of doses administered of the COVID-19 vaccines was over 62 million [2].

Before availability of COVID-19 vaccines, the only effective preventive measure was to avoid exposure. Measures like social distancing, curfew, and mass economic shutdowns resulted in severe deterioration of psychosocial and physical wellbeing and a great decline in global economy [3]. COVID-19 vaccination is the best way to control the pandemic. However, vaccine availability does not necessarily mean successful mass vaccination program. Many challenges might face vaccination including rapid and mass manufacturing, global fair distribution, and financial issues [4].

For a vaccination program to be successful, authorities should consider many factors such as public acceptance, facilities for wide coverage, impact of infodemics, and trust in authorities. In addition, many inquiries surround COVID-19 vaccination including short- and long-term side effects, need for frequent vaccinations, and long-term protection, and its ability to protect against different genetic variants should be considered [5].

Vaccine acceptance may be affected by demographic, behavioral, and health predictors [6, 7]. Vaccine hesitancy refers to delay in accepting or refusing vaccine, although it is available. This issue is not new; however, it has become more apparent with new COVID-19 vaccines and represents a barrier to resolving the crisis. Even people who would usually trust vaccines prefer to wait for more information. Lack of confidence in vaccine safety and effectiveness, low perception of magnitude of disease, and fear of vaccine availability, accessibility, and affordability can be a barrier against its acceptance [8].

A large study analyzing 15 surveys in low- and middle-income countries (LMIC) and Russia and the USA [9] showed that the average acceptance rate across all LMIC studies was 80.3% that was higher than in the USA (64.6%) and Russia (30.4%). The most frequently cited reason for vaccine acceptance was the personal protective benefit of vaccination, whereas concern about side effects was the most frequently cited reason for vaccine hesitancy.

In Saudi Arabia, the acceptance rate among adult Saudi population at different regions ranged from 48 to 65% [10–14]. Limited studies were conducted in Makkah where residents have diverse cultural backgrounds and different levels of exposure that may influence their vaccine acceptance. We aimed to investigate acceptance and trust in COVID-19 vaccines among adult Saudi in Makkah and to explore relationship between their sociodemographics and knowledge level and their acceptance.

## Methods

### Study design and participants

An analytical cross-sectional web-based survey using a snowballing sample was designed. Enrolled participants were adult Saudi population living in Makkah city aged > 18 years who were social media users and agreed to take part in this survey.

### Sample

The minimum sample size required was 385 according to Raosoft sample size calculator based on 95% confidence level, 5% margin of error, and anticipated response of 50%. To reduce the sampling error, the sample size was increased to around 500.

### Study variables

The dependent variables were acceptance rate and trust in effectiveness and safety of COVID-19 vaccines among adult population, while the independent variables (predictors) were sociodemographics and level of knowledge.

### Survey development

An electronic survey was developed for wider and rapid distribution and to reach the target population who conform to regulations of avoiding social contact from 14 to 28 February 2021. The survey was developed in Arabic after a thorough search in the literature and manuscripts with similar aims and questionnaires [3, 5–15]. Initial draft was sent to a group of multidisciplinary experts to validate questions in terms of simplicity, relativity, and clarity. This step was followed by a reliability analysis to determine the internal consistency of the items, and the Cronbach alpha reliability coefficient was 0.72 for knowledge.

A pilot study was conducted on forty participants (excluded from final analysis) to test objectivity and validity of questionnaire or any needed modifications, and it was finalized after a series of group discussions with estimated completion time of about 5–10 min.

The survey was uploaded via Google online survey platform and distributed through different social media platforms within Makkah city. In addition, personal communications helped rapid distribution on the Internet. Participants were able to see the survey and answer the questionnaire by just clicking the relevant link.

### Questionnaire and scoring system

- ▪ The questionnaire included the following: sociodemographics of participants, sources of information about COVID-19, history of seasonal flu vaccination, chronic illness, or any type of hypersensitivity. Participants were asked to indicate if they or one of their family members had previously been exposed to COVID-19 infection.
- ▪ Knowledge about COVID-19 vaccines was assessed using a 9-item MCQ and three-way (yes, no, I do not know) scales. These include hearing about vaccines, free availability, the presence of several types, age restrictions, adverse effects, administration, official registration, place for vaccination, and need for vaccination after recovery. One point was given for correct answers and 0 for incorrect/do not know answers. The total knowledge score was 9 (range 0–9). Participants with scores above 75% (7–9) were considered to have good knowledge.
- ▪ Acceptance of COVID-19 vaccines was assessed by intention to receive vaccines, and they were also asked to indicate confidence in vaccine effectiveness (yes, no, or not sure).
- ▪ Factors that may favor their decision to accept or decline the vaccine were based on 10 items. These include efficacy of vaccines, duration of protection, schedule of vaccination, recommendation by authorities, mode of administration, immediate side effects, long-term negative health consequences, local availability, country of manufacture, and others.

### Ethical approval

It was obtained from the Local Committee for Bioethics and Medical Ethics at Umm Al-Qura University (no. MGNA310121; 14 February 2021). An electronic informed consent from anonymous participants was added as an initial cover page before answering the online survey with emphasis on voluntary participation and withdrawal without justification.

### Statistical analysis

It was carried out using SPSS package (IBM 25.0, Armonk, NY: IBM Corp., USA). Mean ± SD were used for quantitative variables, while frequency and percentage were used for qualitative variables. Chi-square or Fisher exact tests were used to assess differences in frequencies of qualitative variables while Mann-Whitney or Kruskal-Wallis tests were used for continuous nonparametric variables. Logistic regression analysis was used to predict the factors associated with vaccine acceptance and trust. Odds ratios (OR) and 95% confidence intervals (CI) were measured in the univariate analysis, and only significant independent variables were entered in the logistic analysis. Statistical methods were verified, assuming a significant level of < 0.05.

## Results

The study included 507 participants fulfilling inclusion criteria with mean age of 45.2 ± 13.9 years ranged from 18 to 78 years, 55.8% were females, 30% resides in rented houses, 36.7% were married, 86.2% were university students/graduates, about one-third had governmental work, and 29.4% had monthly income less than 5000 SAR. More than one-third (35.3%) had history of previous seasonal influenza vaccination, 12.4% of chronic diseases, 10.1% of any type of hypersensitivity, and 36.7% of participants, or one of their family members had previously been exposed to COVID-19 infection.

The vast majority (99%) of participants heard about COVID-19 vaccines, 97.6% knew that the vaccine is freely available in the kingdom, and 94.3% knew that, at the time of the survey, there were several types of vaccine. The suitable and safe age groups for vaccination were known by 86.2%, vaccines can cause mild side effects, and given by injection were known by 88.2% and 97.2%, respectively. Only 8.5% did not know that they have to register in “Sehhaty” application to get vaccine, more than half of participants (54.8%) did not know the available places for vaccination, ؜while 30.6% did not know that they should take vaccine after recovery from infection (Table 1).

**Table 1:** Knowledge of adult Saudi population in Makkah city about COVID-19 vaccination, 2021

Variables	Frequency (***n*** = 507)	Percent (%)
**Hearing about COVID-19 vaccines**	502	99.0
**Free vaccine availability**	495	97.6
**Several types of COVID-19 vaccines**	478	94.3
**Suitable and safe age groups for vaccination**	437	86.2
**The vaccines can cause mild side effects**	447	88.2
**The vaccines are given by injection**	493	97.2
**Registration via “Sehhaty” application**	464	91.5
**Place for vaccination**	229	45.2
**Vaccination after recovery**	352	69.4

The main sources of their knowledge about COVID-19 were from health officials (59.6%) and social media (59%), while 43% and 32.3% relied on websites and TV, respectively. Only 13% relied on magazines and newspapers.

More than 70% (71.2%) of participants intended to receive COVID-19 vaccination, and 56.4% were confident of vaccine effectiveness. Their level of knowledge about COVID-19 vaccination was satisfactory where 86.2% had good knowledge that was significantly higher with increased age, among those with university education/graduates, governmental workers, those with higher monthly income, had history of previous seasonal influenza vaccination, had or one of their family members previously been exposed to COVID-19 infection, those with intent to receive vaccine, and those who believe in vaccine effectiveness. On the other hand, knowledge level was not affected by sex, lodging type, marital status, or having chronic disorder or any type of hypersensitivity (Table 2).

**Table 2:** Relation between knowledge level and different study variables

Variables	Good knowledge ***n*** = 437 (86.2%)	Poor knowledge ***n*** = 70 (13.8%)	Total ***n*** = 507 (100%)	***p***-value
**Age (years**) mean ± SD	46.0 ± 13.5	40.3 ± 15.2	45.2 ± 13.9	0.001^c^
**Sex**				0.197
Male	188 (43.0)	36 (51.4)	224 (44.2)	
Female	249 (57.0)	34 (48.6)	283 (55.8)	
**Lodging type**				0.121
Rented	137 (31.4)	15 (21.4)	152 (30.0)	
Owned	300 (68.6)	55 (78.6)	355 (70.0)	
**Marital status**				0.143
Married	166 (38.0)	20 (28.6)	186 (36.7)	
Unmarried^a^	271 (62.0)	50 (71.4)	321 (63.3)	
**Educational level**				0.004^c^
Pre-university	52 (11.9)	18 (25.7)	70 (13.8)	
University/graduate	385 (88.1)	52 (74.3)	437 (86.2)	
**Occupation**				< 0.001^c^
Student	197 (45.1)	23 (32.9)	220 (43.4)	
Governmental	156 (35.7)	14 (20.0)	170 (33.5)	
Private	20 (4.6)	14 (20.0)	34 (6.7)	
Not working^b^	64 (14.6)	19 (27.1)	83 (16.4)	
**Family income/month (SAR)**				0.006^c^
< 5000	120 (27.5)	29 (41.4)	149 (29.4)	
5000–10,000	65 (14.9)	15 (21.4)	80 (15.8)	
> 10,000	252 (57.7)	26 (37.1)	278 (54.8)	
**Previous influenza vaccination**	163 (37.3)	16 (22.9)	179 (35.3)	0.022^c^
**Having chronic disorder**	52 (11.9)	11 (15.7)	63 (12.4)	0.434
**Hypersensitivity**	43 (9.8)	8 (11.4)	51 (10.1)	0.670
**History of COVID-19 infection**	171 (39.1)	15 (21.4)	186 (36.7)	0.005^c^
**Intent to receive COVID-19 vaccine**	321 (73.5)	40 (57.1)	361 (71.2)	0.007^c^
**Vaccine effectiveness**	261 (59.7)	25 (35.7)	286 (56.4)	< 0.001^c^

Values presented as number and percent, analyzed by chi-square or Fisher exact tests. Values presented as mean ± SD, analyzed by Mann-Whitney *U*-test

^a^Includes single, widow, and divorced

^b^Includes retired

^c^Significant

**Fig. 1:**
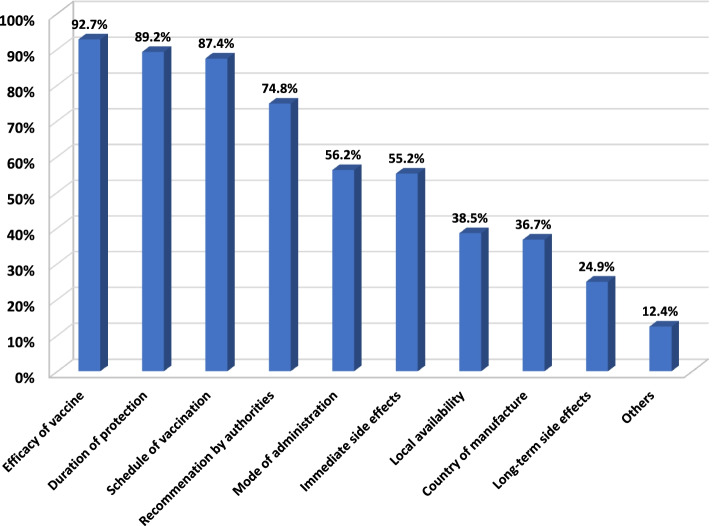
Factors that may influence participants’ decision to accept one of the COVID-19 vaccines

When asked about factors that may favor their decision to accept or decline one of COVID-19 vaccines, the majority reported efficacy of vaccines, duration of protection, and schedule of vaccination (one time vs. multiple times) (92.7%), ( 89.2%), and ( 87.4%), respectively. About three-forths considered recommendation by authorities, and more than half of them considered mode of administration (oral or injection) (56.2%) and immediate side effects (55.2%). Factors less likely to favor their decision were local availability, country of manufacture, and long-term negative health consequences, and others (Fig. 1).

Some sociodemographic factors significantly affect intent to receive vaccines (vaccine acceptance) and trust in vaccine effectiveness including age (higher ages), sex (female), and education (university/graduate). Other significant factors include history of previous seasonal flu vaccination or COVID-19 infection and having good knowledge about vaccines. In addition, those with history of previous seasonal flu vaccination or COVID-19 infection and had good knowledge about vaccines. Higher family income significantly affects participants’ intent to receive vaccines (Table 3).

**Table 3:** Relation between different study variables and vaccine acceptance and trust

Variables	Vaccine acceptance ***n*** = 361 (%)	***p***-value	Trust in vaccine effectiveness ***n*** = 286 (%)	***p***-value
**Age (years**) mean ± SD				
Yes	46.1 ± 13.8	0.027^a^	46.3 ± 14.0	0.036^a^
No/not sure	43.1 ± 14.0		43.7 ± 13.7	
**Sex**				
Male	147 (40.7)	0.018^a^	84 (29.4)	< 0.001^a^
Female	214 (59.3)		202 (70.6)	
**Lodging type**				
Rented	109 (30.2)	0.915	80 (28.0)	0.283
Owned	252 (69.8)		206 (72.0)	
**Marital status**				
Married	127 (35.2)	0.309	109 (38.1)	0.4591
Unmarried	234 (64.8)		177 (61.9)	
**Educational level**				
Pre-university	42 (11.6)	0.033^a^	21 (7.3)	< 0.001^a^
University/graduate	319 (88.4)		265 (92.7)	
**Occupation**				
Student	159 (44.0)	0.230	126 (44.1)	0.866
Governmental	123 (34.1)		98 (34.3)	
Private	27 (7.5)		18 (6.3)	
Not working	52 (14.4)		44 (15.4)	
**Family income/month (SAR)**				
< 5000	97 (26.9)	0.022^a^	102 (28.3)	0.175
5000–10,000	52 (14.4)		52 (14.4)	
> 10000	212 (58.7)		207 (57.3)	
**Previous influenza vaccination**	139 (38.5)	0.018^a^	119 (41.6)	0.001^a^
**Having chronic disorder**	44 (12.2)	0.769	34 (11.9)	0.686
**Hypersensitivity**	33 (9.1)	0.328	30 (10.5)	0.767
**History of COVID-19 infection**	143 (39.6)	0.033^a^	120 (42.0)	0.005^a^
**Good knowledge about vaccines**	321 (88.9)	0.007^a^	261 (91.3)	< 0.001^a^

Values presented as number and percent, analyzed by chi-square or Fisher exact tests. Values presented as mean ± SD, analyzed by Mann-Whitney *U*-test

^a^Significant

Variables that were significantly related to their intent to receive vaccines and trust in vaccine effectiveness were further analyzed by multinomial logistic regression to predict the independent variables contributing to vaccine acceptance and trust. Significant predictors for both vaccine acceptance and trust included good knowledge about vaccines (*OR* = 2.07; *CI*: 1.24–3.48 for acceptance, and *OR* = 2.67; *CI*: 1.58–4.51 for trust), higher educational level (*OR* = 1.80; *CI*: 1.07–3.40 for acceptance and *OR* = 3.59; *CI*: 2.08–6.21 for trust), previous seasonal flu vaccination (*OR* = 1.66; *CI*: 1.09–2.53 for acceptance and = 1.91; *CI*: 1.31–2.79 for trust), female sex (*OR* = 1.62; *CI*: 1.1–2.39 for acceptance and *OR* = 4.15; *CI*: 2.86–6.04 for trust), and history of COVID-19 infection (*OR* = 1.57; *CI*: 1.04–2.37 for acceptance and *OR* = 1.69; *CI*: 1.17–2.46 for trust) (Table 4).

**Table 4:** Regression analysis of factors predicting COVID-19 vaccine acceptance and trust among adult Saudi population in Makkah city, 2021

Independent variables	Vaccine acceptance			Trust in vaccine effectiveness		
	***B***	OR (95% ***CI***)	***p***-value	***B***	OR (95% ***CI***)	***p***-value
Age	0.02	1.02 (1.0–1.03)	0.028^a^	0.01	1.01 (1.0–1.03)	0.036^a^
Sex (female)	0.48	1.62 (1.1–2.39)	0.014^a^	1.42	4.15 (2.86–6.04)	< 0.001^a^
Educational level (university/graduate)	0.59	1.80 (1.07–3.40)	0.027^a^	1.28	3.59 (2.08–6.21)	< 0.001^a^
Family income/month (> 10,000 SAR)	0.29	1.34 (0.87–2.08)	0.187	0.43	1.54 (0.94–2.54)	0.088
Previous influenza vaccination	0.51	1.66 (1.09–2.53)	0.018^a^	0.65	1.91 (1.31–2.79)	0.001^a^
History of COVID-19 infection	0.45	1.57 (1.04–2.37)	0.032^a^	0.53	1.69 (1.17–2.46)	0.005^a^
Knowledge score (good)	0.73	2.07 (1.24–3.48)	0.006^a^	0.98	2.67 (1.58–4.51)	< 0.001^a^

*OR* odds ratio, *CI* confidence interval

^a^Significant

## Discussion

This study aimed to examine the level of acceptance to take the COVID-19 vaccines among adults in Makkah city and to investigate the factors that determine their willingness to be vaccinated. Public behavior towards vaccines –– whether acceptance, refusal, or hesitancy –– may be related to psychological, societal, and vaccine-related aspects (such as safety, side effects, effectiveness, efficacy) that may enhance or hamper herd immunity and are critical for planning effective health communications to encourage mass immunization [16].

### Knowledge about COVID-19 vaccination

The majority of participants (86.2%) had good knowledge about COVID-19 vaccination, and those intended to receive vaccines and had trust in their effectiveness had significantly higher knowledge. Our findings were comparable with other research assessing public knowledge about COVID-19 vaccination: 90.9% in Egypt [17], 79% in Vietnam [18], 78.3% in Libya [19], and 74% in Ethiopia [20]. On the other hand, the overall knowledge level in India was poor as almost half of participants reported incorrect or did not know responses [21], and only 56.6% in Bangladesh reported good knowledge with mean scores of 2.83 ± 1.48 (out of 5) [22].

Good knowledge among participants was significantly related to increased age, high educational and financial levels, governmental workers, those who had history of previous seasonal influenza vaccination, and those who had or one of their family members had previously been exposed to COVID-19 infection. Similarly, Shafiq et al. found that US participants 55 years and older and those with higher educational background reported a higher average COVID-19 knowledge score [23]. Also, Gallè et al. found a significant correlation of knowledge level among Italian with age, educational level, being a HCW, attending a course on life science, and being vaccinated or had intent to be vaccinated against COVID-19. Therefore, outreaching young age and low education and income level groups with community involvement and awareness campaigns are important [24].

The literature on gender and COVID-19 vaccine acceptance is mixed, with most studies indicating higher male acceptance, e.g., in France [25], the UK, and Turkey [26]. However, our findings did not find a gender effect on knowledge level. This may be attributed to comparable male to female ratio in our study, indicating a better sample distribution by gender. Similarly, no gender differences were found among the Malaysian public ([27], preprint) and HCWs in Pakistan [28] in the extent of perceived knowledge or perceived susceptibility of COVID-19 risk factors.

### COVID-19 vaccination acceptance

Intent to receive vaccines was reported by 71.2% of participants with only 56.4% were confident of vaccine effectiveness. This finding is notable considering the potential hesitancy associated with new vaccines.

Our results were higher than those reported among Saudi population in different regions. Al-Mohaithef and Padhi found that, among adult Saudi in major cities (Riyadh, Dammam, Jeddah, and Abha) and other minor cities, 64.7% intended to uptake the hypothetical COVID-19 vaccine, only 7% reported hesitancy, and 28.2% were reported “not sure” about their intention [14]. Alshahrani et al. found that, across the five main Saudi regions (Southern, Northern, Central, Eastern, and Western), 63.9% showed a desire to accept COVID-19 vaccines, while 18.3% were extremely hesitant [13]. Alqahtani found that 57.3% of adult population in the Southern region of Saudi Arabia were willing to receive the new COVID-19 vaccine [12]. In another Saudi study covering Central, Western, and Eastern provinces, 53.3% of public have shown intent to be vaccinated, 78.8% were at high risk of COVID-19, and only 46.6% had trust in healthcare system [11]. Alfageeh et al. found that those willing to receive COVID-19 vaccines were reported by 48% of Saudi adults across the five main Saudi regions [10].

The higher rate of vaccine acceptance among Makkah population may be attributed to population dynamics (specially with the habitancy of diverse multicultural population in Makkah city for decades), literacy levels, experience in management of vaccine preventable disease (particularly with Hajj and Umrah), and other factors. In addition, “intervention fatigue” among the community (from prolonged compulsory mask wearing laws, nationwide lockdowns, and curfews that have been strictly implemented since the beginning of the pandemic) could be a possible explanation. However, this has not been studied and needs further evaluation.

In nearby Arab countries, variable levels of public acceptability of COVID-19 vaccines were noticed with higher willing in Iraq (88.6%) [29] and Egypt (88%) [17], moderate in Kuwait (67.3%) [30] and the United Arab Emirates (60.1%) [31], and it was fairly low (37.4%) in Jordan [32].

A large-scale multinational study measured vaccine hesitancy among Arab-speaking subjects (*n* = 36,220) from all the 23 Arab countries and territories (*n* = 30,200; 83.4%) and Arabs who live in 122 other countries (*n* = 6020; 16.6%) including Europe, North America, Turkey, and others. A significant rate of vaccine hesitancy was reported among Arabs in and outside the Arab region (83% and 81%, respectively). The most cited reasons for hesitancy were concerns about side effects and vaccine safety, distrust in health care policies, and vaccine expedited production [33].

Wouters et al. [34] examined the potential acceptance of COVID-19 vaccines in 32 countries. Acceptance was highest in Vietnam (98%), China and India (91%), and South Korea and Denmark (87%) and lowest in Paraguay (51%), France and Lebanon (44%), Croatia (41%), and Serbia (38%). Another global survey from 19 countries found that 71.5% of participants reported being very or somewhat likely to take a COVID-19 vaccine with heterogeneous responses between countries as highest acceptance (90%) was reported in China, while Russia reported the least (55%) [35]. The variations in vaccine acceptance rates between studies may be explained by social, cultural, political, or economic differences between countries [36]. Furthermore, the time of conducting the surveys in relation to the pandemic phase and the prevailing variant of the virus may be a key factor explaining these variations.

Vaccine efficacy, duration of protection, and schedule of vaccination were among the essential factors that may favor the decision of our participants to accept the vaccines. Kreps et al. ascribed such an attitude of acceptance among US adults to certain factors including vaccine efficacy, major and minor adverse events, duration of protection, approval process, country of origin, and political ratification. In addition, history of influenza vaccination, contact with severe cases, and attitudes towards vaccine safety could explain variation in vaccination hesitancy among different groups [37].

Public concern about vaccines safety and efficacy has been reported as major barrier to vaccination decision-making, especially for newly developed vaccines. In fact, actual uptake of pandemic vaccines might be lower than acceptance after beginning of mass immunization [38]. This may be attributed to low-perceived susceptibility and high-perceived barriers to vaccination such as physical, psychosocial, or financial barriers [39]. At the end of domestic H1N1 outbreak in France, only 10% of population received a vaccination compared to vaccination intention of 17% [38]. However, some research found a considerable consistency level between self-reported acceptance and actual uptakes [40, 41].

In addition, vaccine acceptance may be changed in different epidemic phases as monitored by Wang et al. in China where intention to accept immediate vaccination after availability has declined substantially, from 52.2% in Mar 2020 to 24.7% in Nov–Dec 2020 due to concerns about vaccine safety [42].

### Predictors of vaccine acceptance and trust

Vaccine acceptance and trust were affected by level of knowledge about vaccine and some sociodemographic factors. Studies showed similarities and differences regarding these factors with different explanations [43, 44]. Age, gender, education, occupation, or income were reported [43]. Also, lack of vaccine acceptance was found to be associated with conspiratorial theory, lack of trust in authorities, lack of concern about COVID-19, and susceptibility to misinformation around COVID-19 [44].

Lennon et al. found conflicting results with traditional sociodemographic factors, political views, and religiosity did not predict vaccine intentions among US adults living in a rural college town [45]. Similarly, Del Riccio et al. found age, level of education, and knowing infected or hospitalized relatives were not predicting vaccine acceptance in a population-based sample in Italy [46]. In some poor communities, traditional remedies, fear of injections, overlapping local terms for vaccine, religious beliefs, and a background of distrust towards western medicine antagonized vaccine acceptance [47].

Planning awareness campaigns with extensive media coverage, promoting benefits of vaccination, providing clear information about vaccines safety and efficacy, and highlighting number of people getting vaccinated, along with testimonials, can motive vaccination process. In addition, official promotion strategies should be based on realizing current public acceptance to ensure fair distribution of COVID-19 vaccines for the public [35].

### Study limitations

It was difficult to establish causal inferences with cross-sectional design; snowball method did not result in random sampling of general population with sambling bias; use of web-based survey may lead to a potential selection bias, underestimation of current situation, and being accessible to web users only; possible overrepresentation of health-oriented and more concerned individuals; recall bias of self-reported information with social desirability bias; and findings might be nonrepresentative of current situation as vaccination acceptance and trust may change with pandemic progress and actual intention could be changed with availability of vaccines or when data about certain vaccines change over time. Furthermore, findings may vary in other populations with different ethnic, cultural, and geographical backgrounds.

## Conclusions

Our results showed satisfactory knowledge about COVID-19 vaccination (86.2%) with moderate rate of vaccine acceptance (71.2%) and a relatively low rate of trust in vaccine effectiveness (56.4%) among adults living in Makkah. Factors as vaccine efficacy, duration of protection, schedule of vaccination, and recommendation by authorities favored their decision to accept or decline vaccines. The strongest predictors for vaccine acceptance and trust were good knowledge about vaccines, higher educational level, previous seasonal flu vaccination, female sex, and history of COVID-19 infection. Our findings can help to design larger nationwide representative studies of Saudi population to better understand public acceptance and trust of COVID-19 vaccines, to handle the issue of vaccine hesitancy, and to address barriers to vaccination in order to improve vaccine coverage among the population and to reinforce some communication characteristics of current vaccination campaigns.

## Data Availability

The datasets used and analyzed during the current study are available from the corresponding author on reasonable request. Confidentiality and security of data and materials were insured through all stages of the study.

## Abbreviations

CI: Confidence intervals
HCW: Healthcare worker
KSA: Kingdom of Saudi Arabia
OR: Odds ratios
UK: United Kingdom
US: United States
WHO: World Health Organization
